# Supplementary figures and images for: Social familiarity strengthens neural and vocal responses to conspecific calls in zebra finches
Source: PLoS Comput Biol. 2026 Mar 11;22(3):e1014024. doi: 10.1371/journal.pcbi.1014024 (PMC12978450; doi:10.1371/journal.pcbi.1014024)

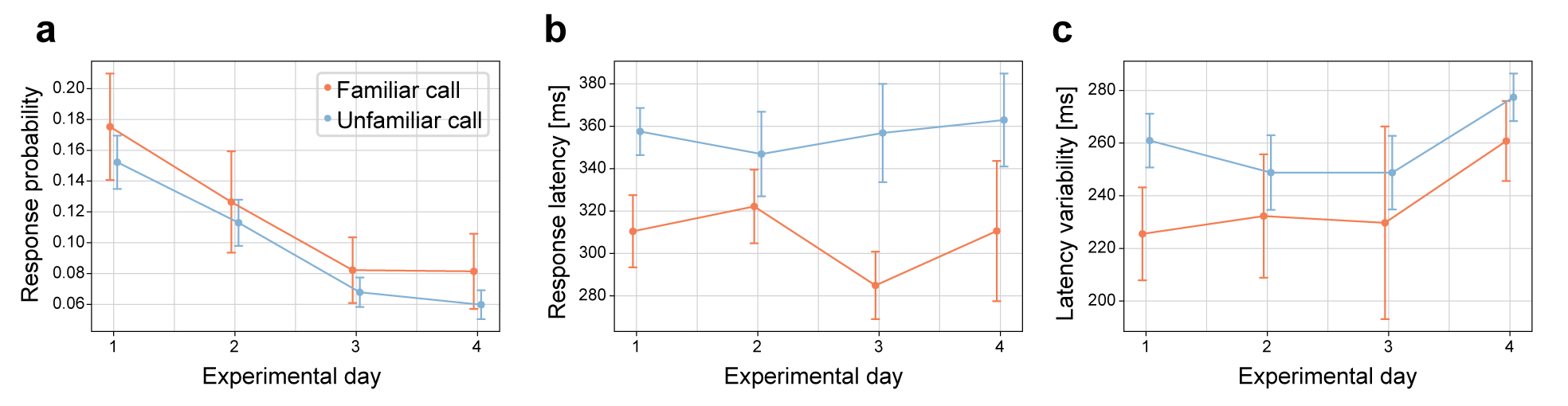

Supplement: S1 Fig — (a) Mean values for peak response probabilities to familiar and unfamiliar call playbacks by experimental day (n = 9, birds = 7). ANOVA, familiarity effect p = 0.003, day effect p = 0.001, interaction p = 0.68. (b, c) Same arrangement as in a, but for response latencies and latency variability. Response latency ANOVA, familiarity effect p = 0.002, day effect p = 0.41, interaction p = 0.49. Response variability ANOVA, familiarity effect p = 0.04, day effect p = 0.18, interaction p = 0.86. Error bars depict standard error. (TIF) [file pcbi.1014024.s001.tif]

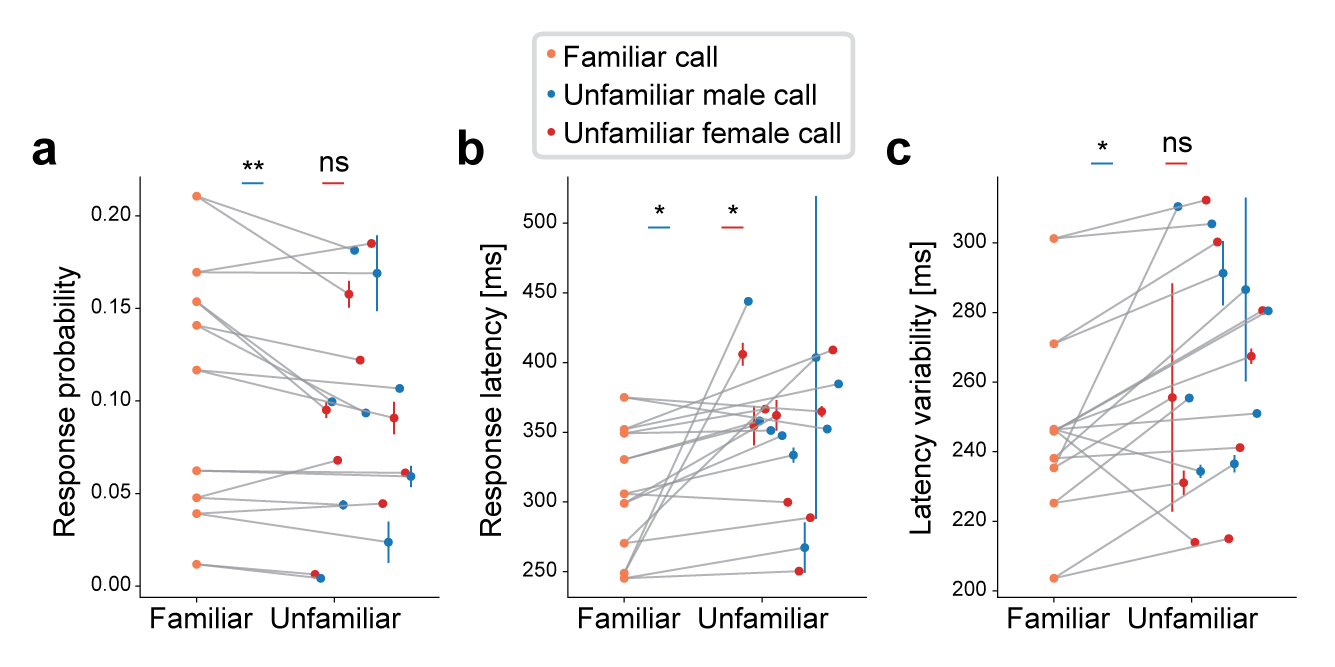

Supplement: S2 Fig — (a) Peak response probabilities to different call playbacks (n = 9, birds = 7, days = 4). Response probability (familiar)=0.117, (unfamiliar male)=0.094, (unfamiliar female)=0.091. Wilcoxon signed-rank test, familiar versus unfamiliar male, p = 0.003. Familiar versus unfamiliar female, p = 0.25. (b, c) Response latencies and response latency variability. Response latency (familiar)=306ms, (unfamiliar male)=352ms, (unfamiliar female)=362ms. Wilcoxon signed-rank test, familiar versus unfamiliar male, p = 0.019. Familiar versus unfamiliar female, p = 0.039. Latency variability (familiar)=246ms, (unfamiliar male)=280ms, (unfamiliar male)=256ms. Wilcoxon signed-rank test, familiar versus unfamiliar male, p = 0.019. Familiar versus unfamiliar female, p = 0.097. Blue solid dots represent mean values across multiple unfamiliar playbacks when applicable, and error bars standard error (sem). * denote p < 0.05, ** p < 0.01, and ns non significant. (TIF) [file pcbi.1014024.s002.tif]

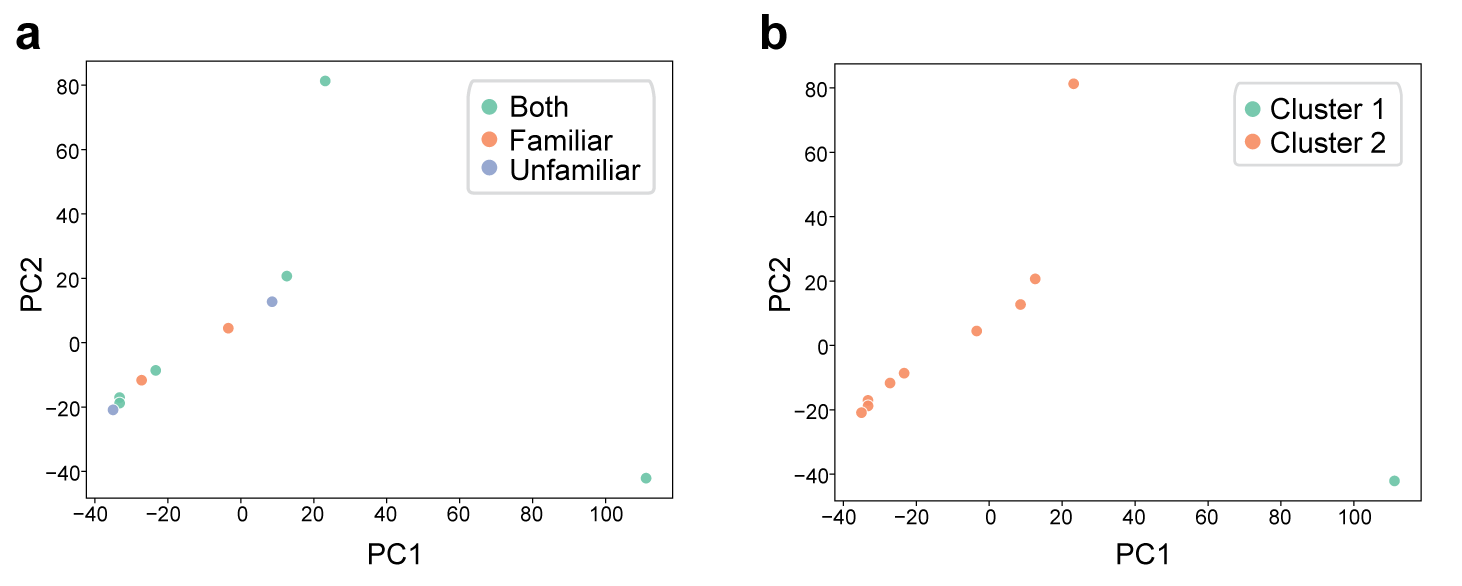

Supplement: S3 Fig — (a) Playbacks projected into principal component space based on their acoustic waveforms (10 playbacks, variance explained by first 2 PCs = 51.03%). Each point represents a single sound. Dots labeled to indicate if playback was used as a familiar, unfamiliar or both during the experiments. (b) Same arrangement as in a, but points are labeled according to cluster id. Using silhouette score, we determined the optimal number of clusters, and identified them using kmeans (see Methods). Only one playback was assigned to Cluster 1 (bottom right), while all other stimuli were labeled as Cluster 2. (TIF) [file pcbi.1014024.s003.tif]

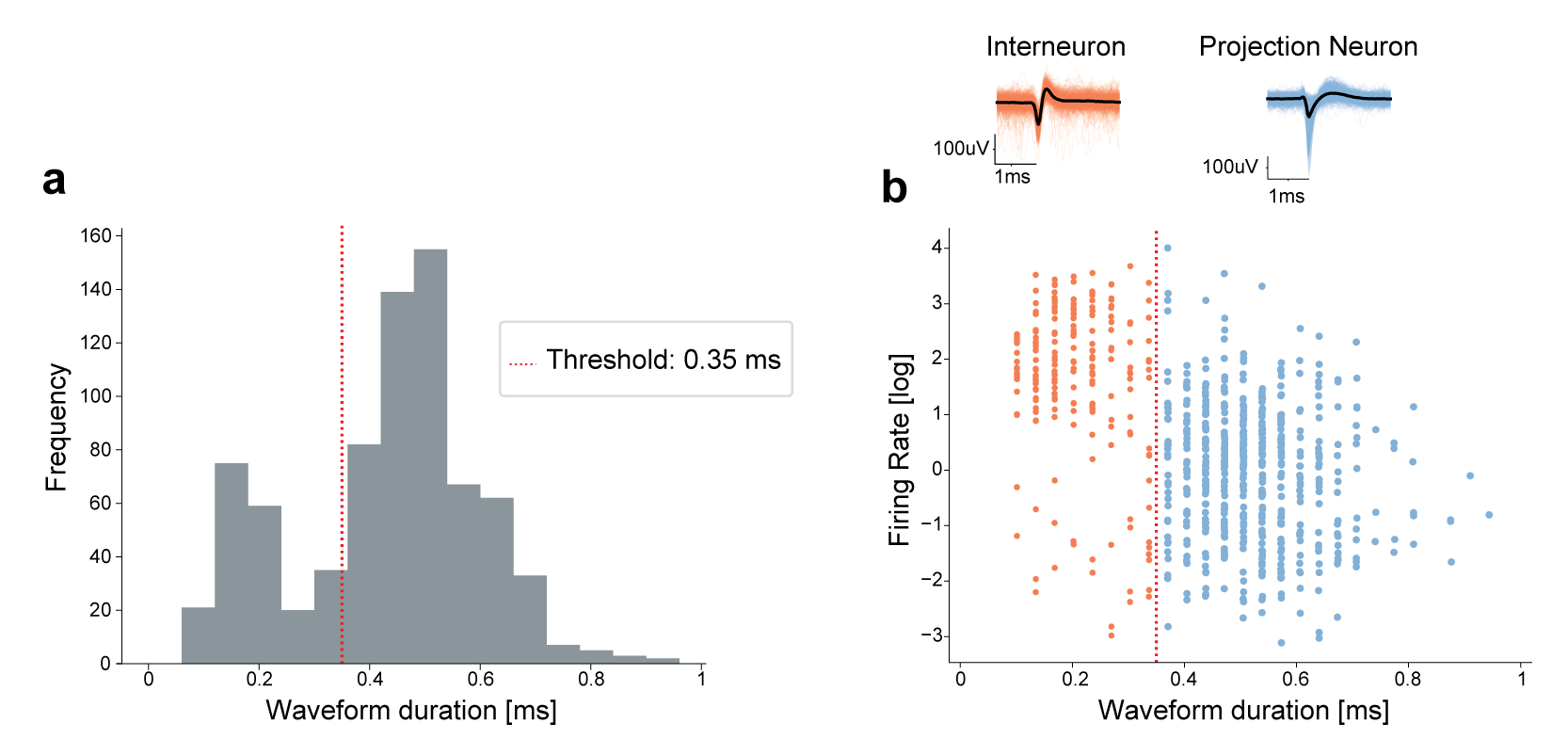

Supplement: S4 Fig — (a) Histogram of trough-to-peak duration for all HVC cells recorded (neurons: 765, recordings = 9, birds = 8, bin size = 0.06ms). Mean interneuron waveform duration = 0.20ms. Mean projection neuron waveform duration = 0.52ms. (b) Top: Example putative interneuron and projection neuron. For each cell, a subset of 1000 spikes were selected randomly and used in the stack traces plotted. The solid black line represents the mean trace. Bottom: Scatterplot of waveform duration and spontaneous firing rate for cells shown in a. Each solid dot corresponds to 1 neuron. Mean firing rate for interneurons: 9.51 ± 8.37Hz. Mean firing rate for projection neurons: 1.93 ± 3.8Hz. Red dashed line indicates the threshold chosen to separate narrow and broad spiking neurons (interneurons = 210, projection neurons = 555). (TIF) [file pcbi.1014024.s004.tif]

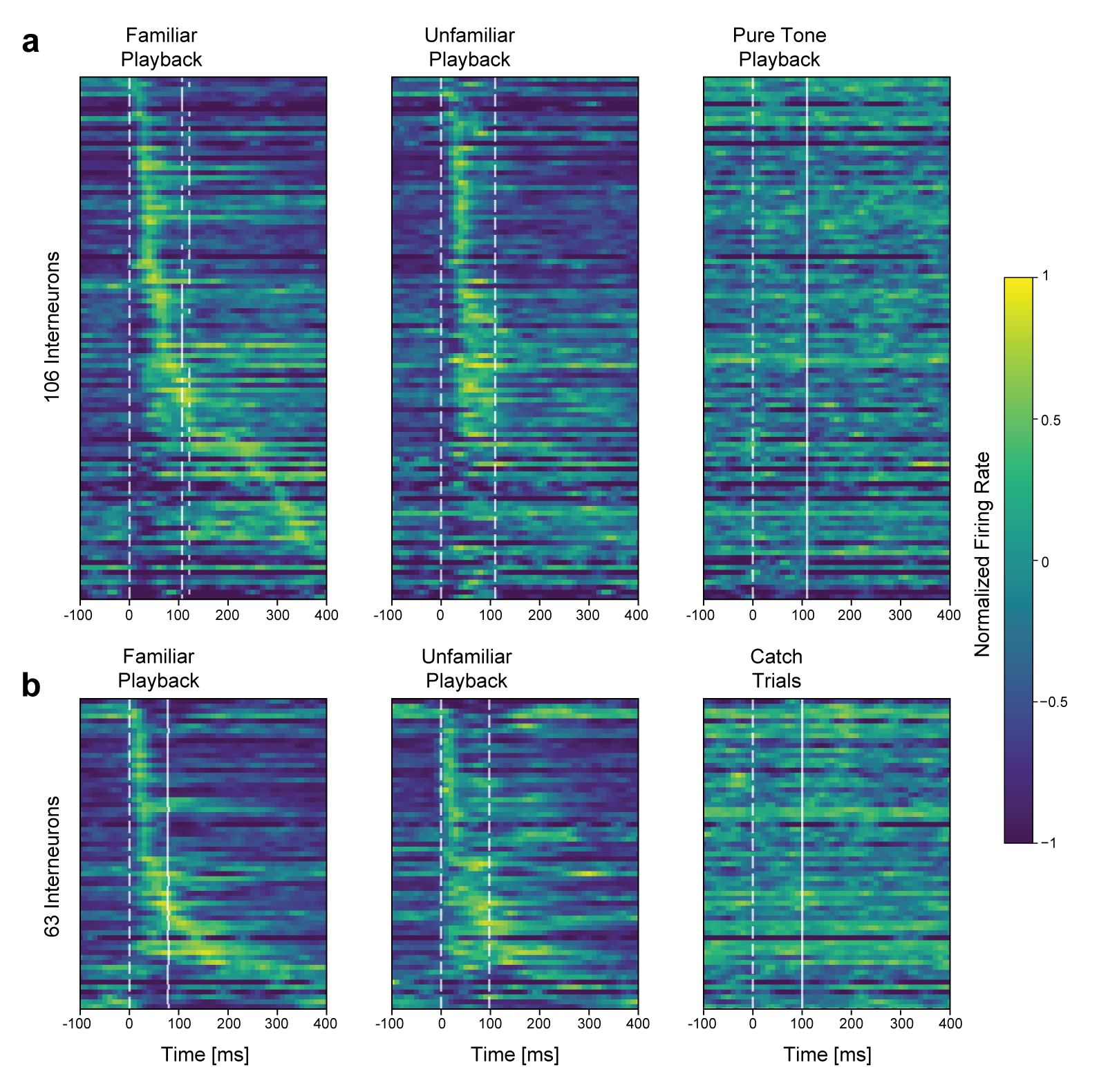

Supplement: S5 Fig — (a) Normalized firing rate for interneurons deviating 2 standard deviations from baseline during the familiar and unfamiliar call playback (recordings = 5, birds = 5). Neurons are ordered by their peak firing time during familiar call playback. The same neuron order is maintained for the other stimuli, showing corresponding activity patterns across conditions. White dashed lines depict call onsets and offsets. The pure tone represents a 20kHz sound presented as a control. (b) Same arrangement as in a), but for a different set of birds (recordings = 4, birds = 3) presented with catch trials (trials with silent playback). (TIF) [file pcbi.1014024.s005.tif]

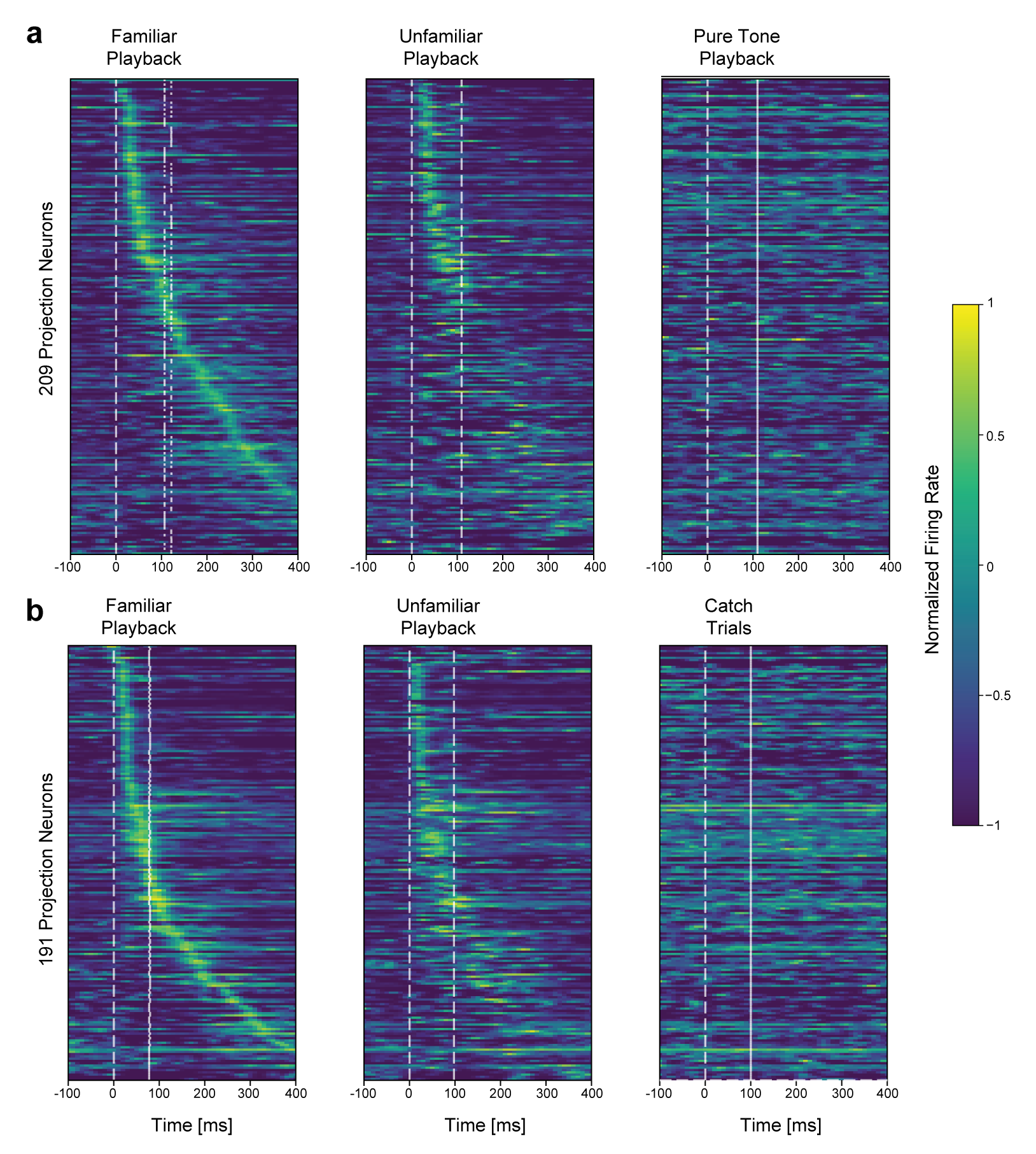

Supplement: S6 Fig — (a) Normalized firing rate for projection neurons deviating 2 standard deviations from baseline during the familiar and unfamiliar call playback (recordings = 5, birds = 5). Neurons are ordered by their peak firing time during familiar call playback. The same neuron order is maintained for the other stimuli, showing corresponding activity patterns across conditions. White dashed lines depict call onsets and offsets. The pure tone represents a 20kHz sound presented as a control. (b) Same arrangement as in a), but for a different set of birds (recordings = 4, birds = 3) presented with catch trials (trials with silent playback). (TIF) [file pcbi.1014024.s006.tif]

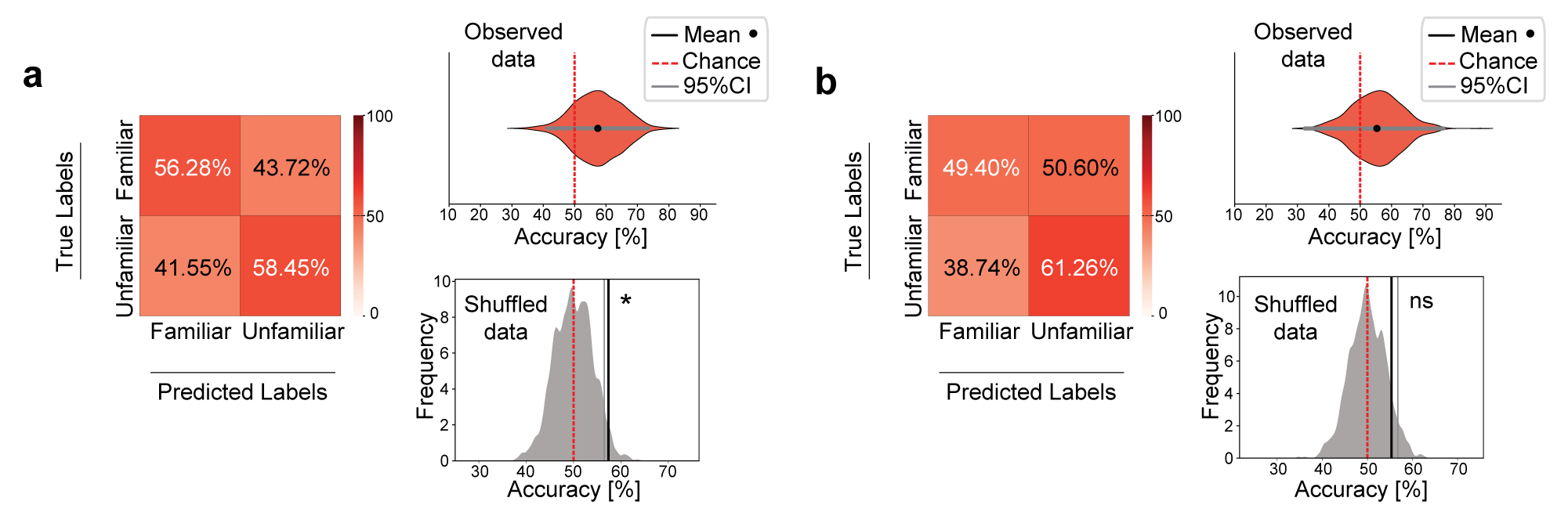

Supplement: S7 Fig — (a) Average classification accuracy for call playback familiarity based on the firing rate of neurons shown in Fig 2b (time window = 0–100ms from playback onset, model = support vector machine, iterations = 1000, test size = 0.1). Left: Confusion matrix. Top Right: Distribution of accuracies across runs (57.36 ± 7.54%). Bottom Right: Kernel density estimate distribution derived from shuffled data. The solid gray line indicates the 95% confidence interval of the shuffled distribution (56.47%), while the black solid line represents the mean accuracy of the observed data (57.36%, permutation test, p = 0.03). When including only during-playback responses (0–100ms), the classifier predicted which call was presented with an accuracy significantly higher than chance. Chance level = 50%. (b) Same as in a, but for the time window = 100ms to 400ms, corresponding to the post-playback period. Top Right: Distribution of accuracies across runs (55.32 ± 7.94%). Bottom Right: 95% confidence interval of the shuffled distribution (56.76%). Mean accuracy of the observed data (55.32%, permutation test, p = 0.085). When including only the late responses (100–400ms), the classifier was unable to reliably predict which call was presented. Chance level = 50%. * denotes p < 0.05 from permutation test, while ns non-significant. (TIF) [file pcbi.1014024.s007.tif]

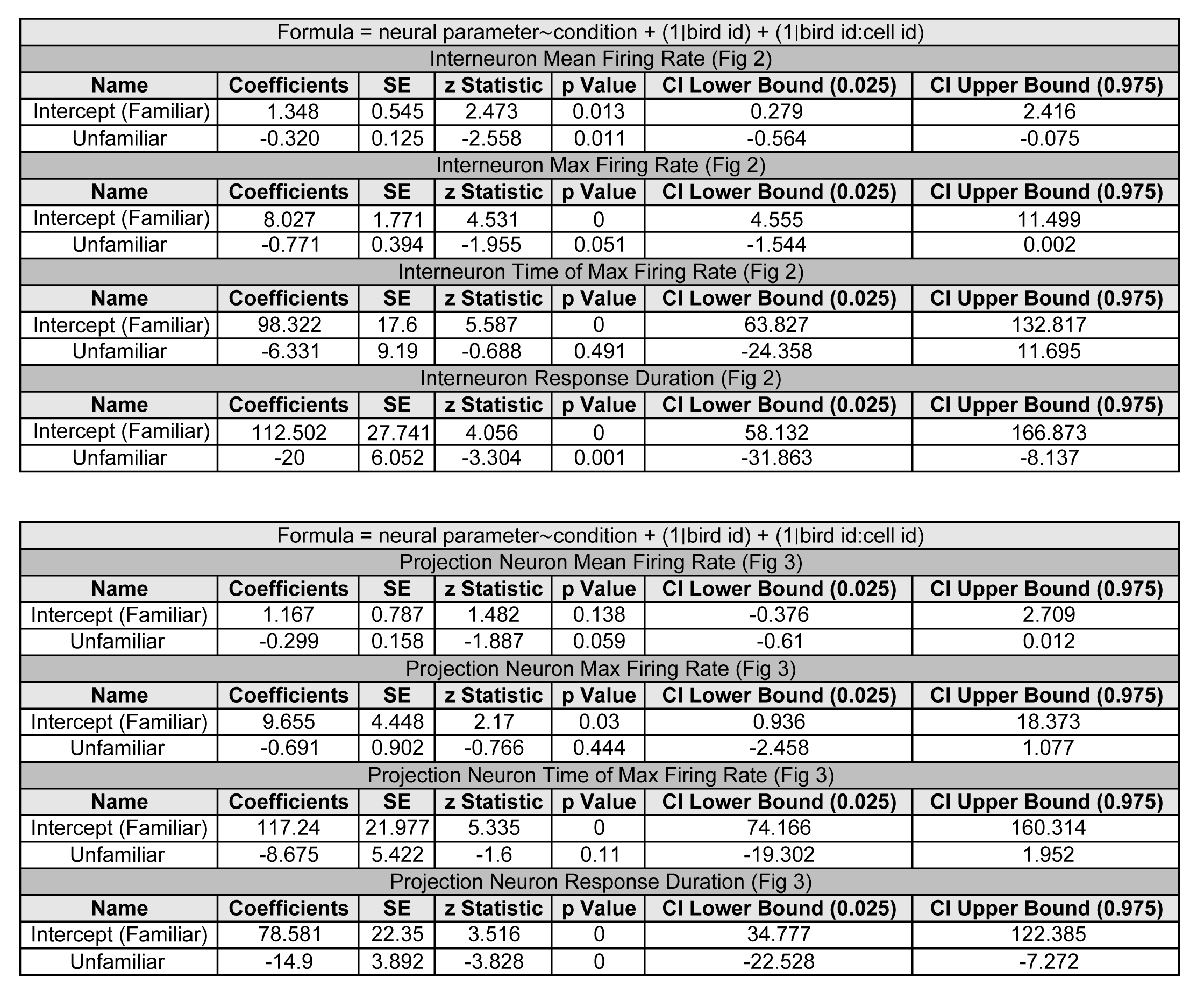

Supplement: S8 Fig — (TIF) [file pcbi.1014024.s008.tif]

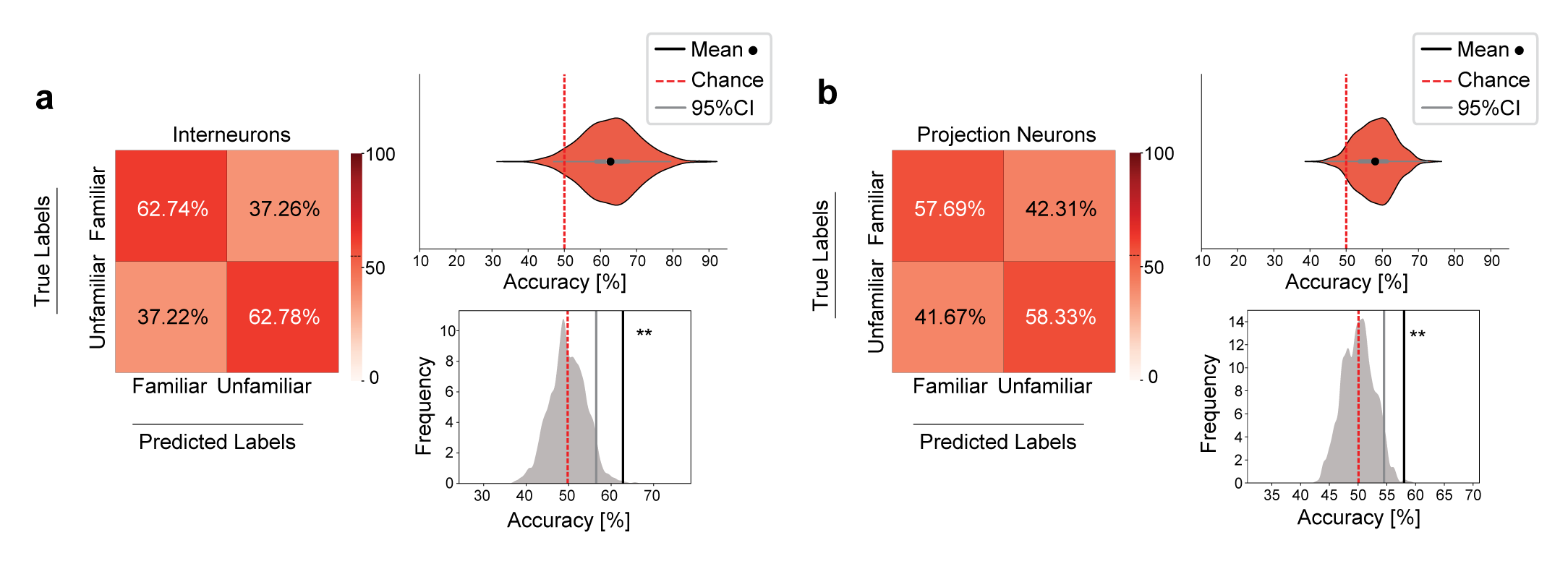

Supplement: S9 Fig — Average classification accuracy to classify call playback familiarity based on neural features that differed for familiar and unfamiliar playbacks (See Fig 2f & 3f) Model = support vector machine, iterations = 1000, test size = 0.1. (a) Familiarity classification using interneurons and based on neural features mean firing rate, max firing rate, and response duration. Left: Confusion matrix. Top Right: Distribution of accuracies across runs (62.76 ± 7.9%). Bottom Right: Kernel density estimate distribution derived from shuffled data. The solid gray line indicates the 95% confidence interval of the shuffled distribution (56.47%), while the black solid line represents the mean accuracy of the observed data (62.76%, permutation test, p = 0.002). Chance level = 49.76%. (b) Same arrangement as in a), but for projection neurons and with a classifier based on neural features mean firing rate and response duration. Observed data mean = 58.01 ± 5.22, 95%CI of shuffled data = 54.51%, permutation test, p = 0.002. Chance level = 50%. (TIF) [file pcbi.1014024.s009.tif]

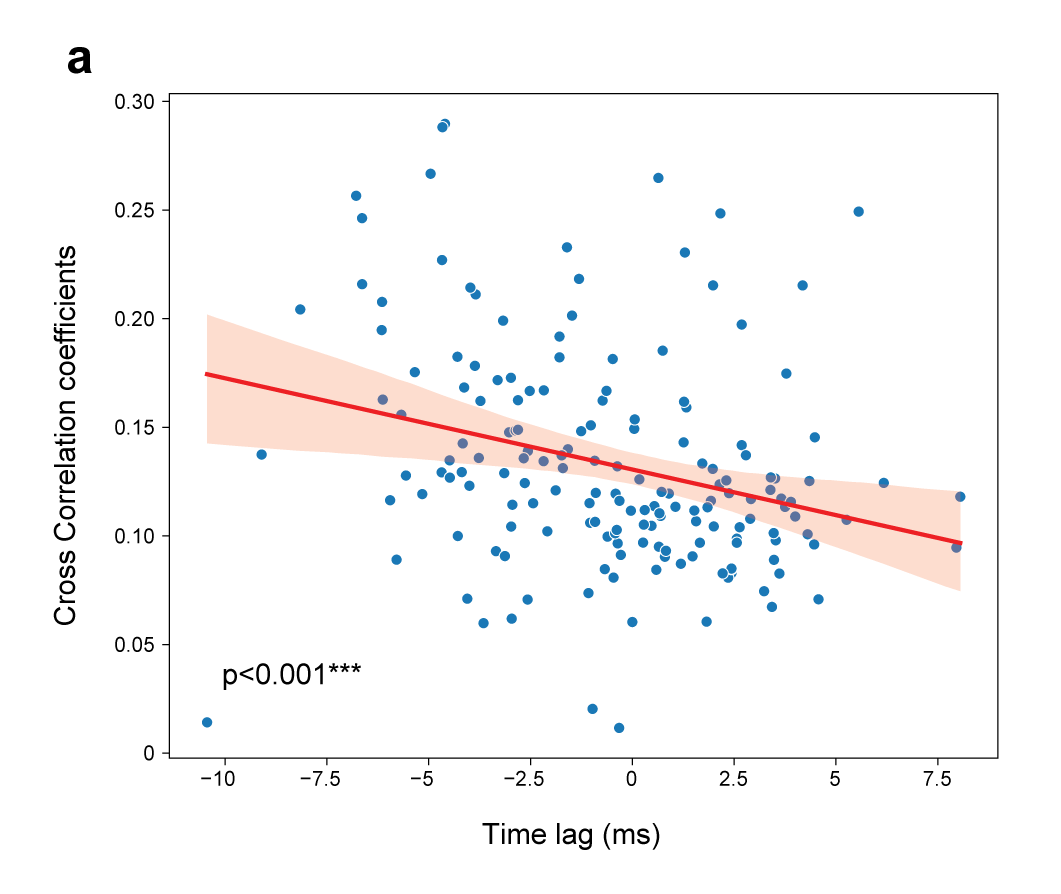

Supplement: S10 Fig — (a) Scatterplot of mean correlation coefficients and mean time lags for all interneurons with auditory-evoked activity recorded (recordings = 8, birds = 9, interneurons = 169, projection neurons = 400). Each dot represents an interneuron and how it correlates on average with all other simultaneously recorded projection neurons and at what time lag. Red line represents fitted regression line and shaded region shows the 95% confidence interval for the regression estimate. *** denote p < 0.001 from Spearman correlation analysis (correlation coefficient = -0.34, p = 7.2 × 10 ⁻ ⁶). (TIF) [file pcbi.1014024.s010.tif]

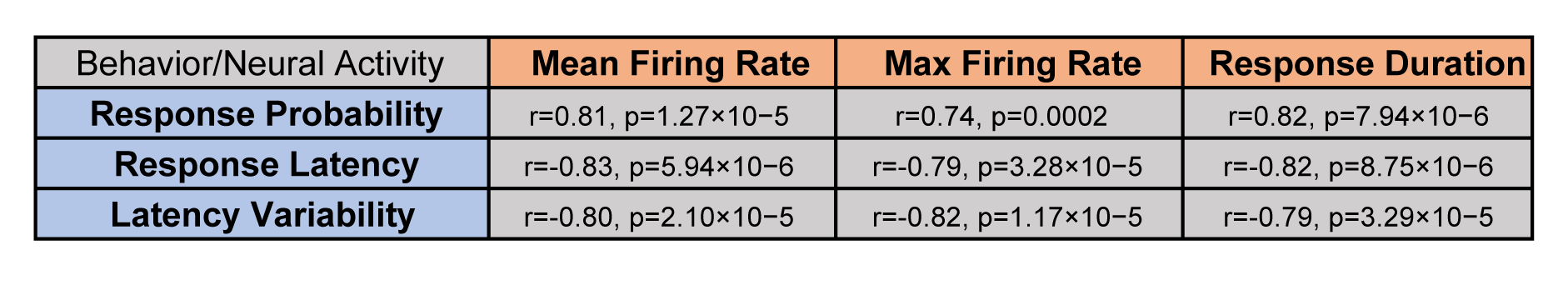

Supplement: S11 Fig — Kolmogorov-Smirnov test used to test for normality, then Pearson correlation coefficient calculated. Bonferroni correction used to control for multiple comparisons. (TIF) [file pcbi.1014024.s011.tif]

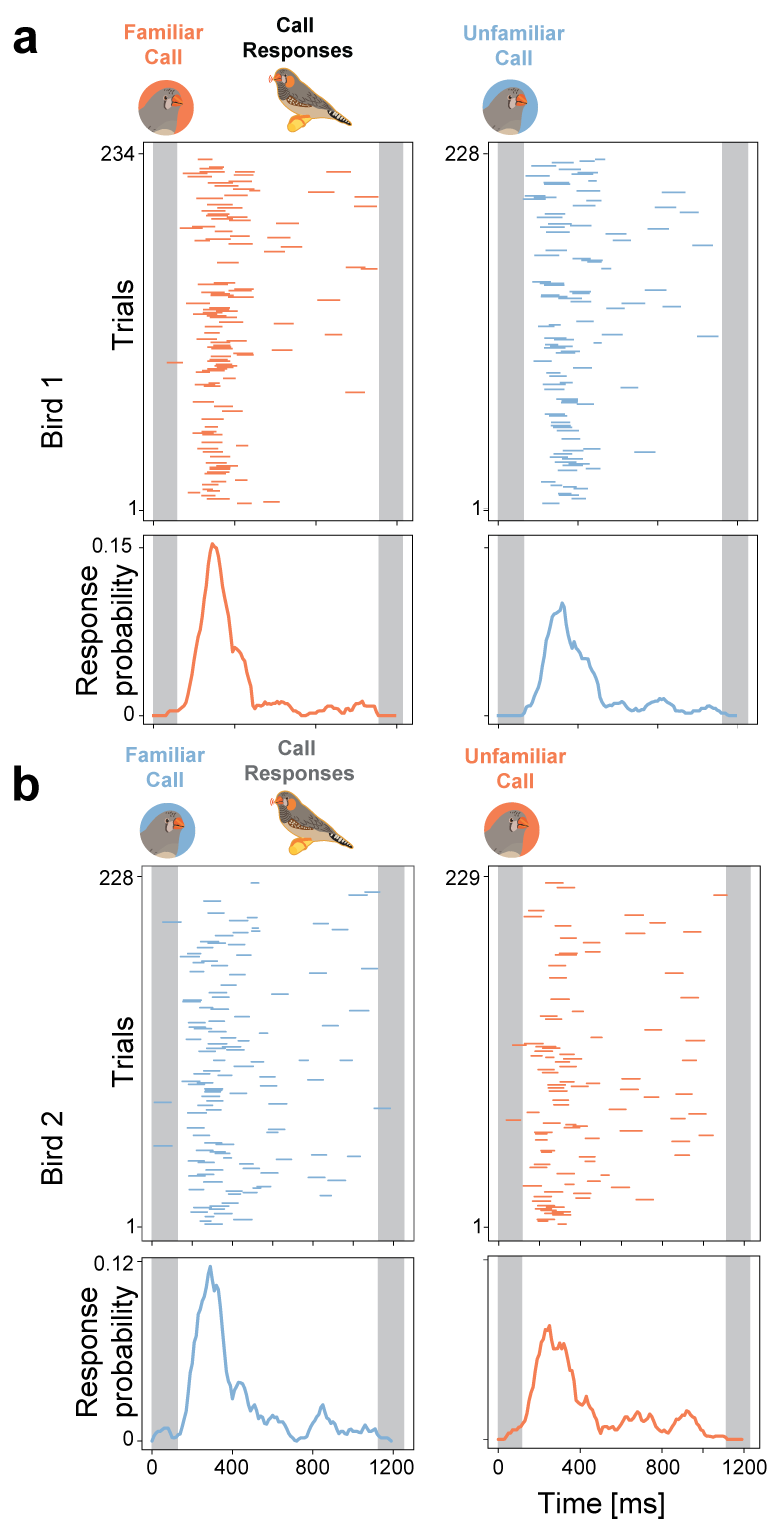

Supplement: S12 Fig — Response profiles from two example birds presented with a familiar and unfamiliar playback. Playback identity is color coded. (a) Top panel: Call responses to call playbacks presented once per second (exemplified by data from the initial two days). Bottom panel: Call response probability (across four days). (b) Same arrangement as in a, but for a different experimental bird. (TIF) [file pcbi.1014024.s012.tif]
