# Supplementary material for: Social familiarity strengthens neural and vocal responses to conspecific calls in zebra finches
Source: PLoS Comput Biol. 2026 Mar 11;22(3):e1014024. doi: 10.1371/journal.pcbi.1014024 (PMC12978450; doi:10.1371/journal.pcbi.1014024)
Supplement: Attachment — Submitted filename: Rebuttal_PlosCompBio_12092025_final.pdf [file pcbi.1014024.s014.pdf]

Responses to reviewer comments (in green):

Reviewer #1: This manuscript makes an important contribution to our understanding of how social familiarity modulates vocal communication circuits in songbirds. The authors have elegantly combined behavioral paradigms with cutting-edge neural recording techniques to reveal how the HVC encodes socially relevant information that guides context-dependent vocal responses.

The authors find that call familiarity leads to more and faster behavioral responses. Correlated with these behavioral findings, HVC interneurons (and to a lesser extent, projection neurons) respond more strongly and persistently, providing a potential neural correlate for the behavioral effects.

This work represents a significant advance in the field of communication neuroscience and merits publication in PLOS Computational Biology.

I have only one minor suggestion for improvement: The increase in response persistence for familiar calls is particularly interesting. Could the authors speculate on potential mechanisms underlying this increased persistence in the discussion section?

We sincerely thank Reviewer #1 for their positive comments and suggestions.

Interestingly, during familiar call playbacks we observed a longer persistent increase in interneuron activity compared to unfamiliar playbacks. The finding that zebra finches respond more quickly to familiar conspecifics would then seem to be at odds with a putative role of inhibitory interneuron activity in withholding calls [8, 21]. However, we speculate that, because these birds were head fixed and did not actively engage in vocal turn taking, the observed differences in interneuron activity might reflect a relatively stronger activation of the withholding mechanism to counter a stronger vocal response drive for familiar calls. We added relevant discussion about this in lines 353-356.

Reviewer #2: A critical feature of social brains is the ability to process social and contextual information and use it to guide appropriate behavioral responses. Understanding how the brain tackles these types of tasks and determines these behavioral decisions remains poorly understood. In this paper, Gomez-Guzman et al. use a clever and simple behavioral paradigm, measuring call responses to call playback, of both familiar and unfamiliar individuals, to probe how population neural responses in a well characterized audio-vocal brain structure code both for perceived call type as well as call initiation and production. They provide convincing behavioral findings that call responses to playback (e.g. latency, probability of response) can be distinguished between the two classes of stimuli (familiar vs. unfamiliar calls). These data are then followed by neuropixel recordings in nucleus HVC showing that call type (familiar vs unfamiliar) can be decoded by the population activity recorded in HVC and that these response properties can then in turn be used to predict when and if the bird will respond with a call. These findings show in this key vocal control area that the brain can very rapidly decode communication signals based on social familiarity and use this information to initiate context appropriate behavioral responses. This is a well written manuscript with generally well-designed experiments.

We thank Reviewer #2 for their favorable assessment of our work.

Primary concern: My only major concern is for the analyses the authors perform in figure 5 to investigate whether HVC auditory evoked responses can be decoded to predict call response probability and latency. producing a call. My concern lies in the potential overlap between the auditory evoked response and premotor activity associated with the produced call. The authors should provide evidence that there is no overlap between sensory and motor activity and some form of validation that these are clearly dissociated in time.

As no Fig 5 is present, we infer that this comment intends to refer to Fig 4. We thank Reviewer#2 for raising an important point and apologize for being unclear in the description of this Figure. While we performed the analysis to correlate the neural response with the behavioral output, it is not a causal relationship. The response rates acquired from the behavioral experiments provide the response probabilities for each bird which were then plotted against neural responses for those birds while only listening. Behavioral measures were taken while birds freely moved in a sound box, while neural recordings were conducted in head-fixed conditions where birds do not readily call back (we did not

conduct simultaneous acquisition of behavior and neural activity). While this paradigm does not strictly rule out an influence of “sub-threshold” premotor drive (insufficient to initiate vocalization while head-fixed; see response to Rev. 1), we initially assumed there would be no overlap between sensory and production-related motor activity. However, your comment inspired us to re-evaluate our dataset. We identified a small number of cases where the bird did call back while head-fixed, and we were intrigued by this finding. We have analyzed this smaller dataset separately, which also allowed us to address your next point.

In addition to this, we added information in the methods to further clarify how experiments were conducted (see lines 505-509).

If there is overlap, then the authors should at least provide evidence that decoding of the auditory response is not biased by underlying pre-motor preparatory activity in HVC, since this could bias their findings. They should also include a raster plot where activity is aligned to the onset of call playback and a parallel raster where activity is aligned to onset of the produced call.

We conducted the electrophysiological experiments in non-habituated, head-fixed birds. In this state, zebra finches are typically unresponsive to audio playbacks. We chose this approach because we were specifically investigating auditory-evoked activity in the HVC, and we wanted to avoid any potential biases from motor activity.

Upon re-evaluating our dataset, we discovered that 4 out of the 8 birds (4/9 recordings) occasionally responded to the call playbacks.

- Bird 1 (M1268): Responded to familiar female playback in 2 out of 60 trials.
- Bird 2 (M1293): Responded to familiar female playback in 7 out of 100 trials.
- Bird 3 (M1109): Responded to familiar female call playbacks in 5 out of 60 trials.
- Bird 4 (M373): Showed responses to multiple playbacks, including familiar female (1/48), unfamiliar female (3/48), and two different unfamiliar male playbacks (1/48 each).

To avoid any overlap between motor and sensory activity, we have now excluded all trials with a vocal response from our analysis. **We have re-analyzed the remaining dataset, and all figures and data in the manuscript have been updated to reflect this change. The main findings of the experiments remain unchanged.** The number of neurons with significant auditory-evoked activity changed marginally from 172/210 (with response trials) to 169/210 (without response trials).

For the reviewers' assessment, we have also provided examples of neurons that illustrate the differences between auditory-evoked and production-related activity (Reviewer Fig 1). Our future research aims to better understand the interaction between these two types of activity within the same neuron. However, we believe this complex question falls outside the scope of the current manuscript.

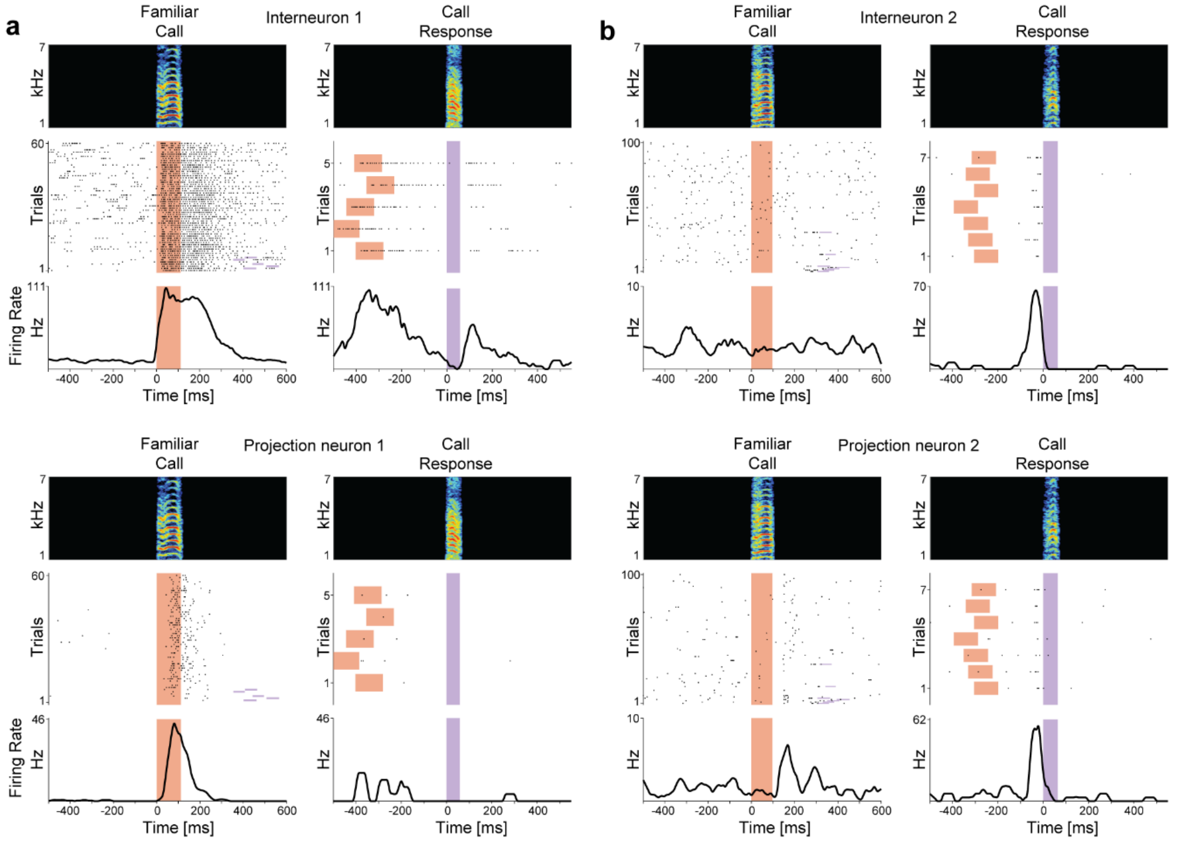

Reviewer Fig 1. Auditory-evoked and motor-related activity in HVC neurons. (a) Example cells with auditory-evoked activity recorded during the presentation of a familiar playback and when bird vocally responded to them. Left side aligned to playback, and right side to vocal responses. Colored rectangles show when call responses (purple) or playbacks (orange) occurred. **(b)** Same arrangement as in a, but in this case neurons that exhibit premotor activity. Each example neuron plotted follows this order: Top: Spectrogram of call playback or response. Middle: Spike dot raster plot. Bottom: peri-stimulus time histogram (PSTH).

Other comments:

1. The authors do a nice job determining neural trajectory in their head fixed recordings in response to call presentations. It is unclear, why they do not perform the same types of analyses in figure 5 for their call back paradigm, instead relying only on features such as neural response duration and maximum and mean firing rate for their analyses? For these experiments, it is also not clear whether these animals are still head fixed and calling or tethered. This should be made clearer in the results as well as the methods section.

Fig 4 aims to demonstrate how call responses are related to neural responses. Therefore, we decided to focus on discrete parameters - which were used to measure both behavior and neural activity – as the basis for assessing their relationship. The neural trajectory analysis we used in Figs 2 & 3 only quantify the differences between responses to different playbacks. During all experiments including electrophysiological recordings, the birds were head-fixed, thus less likely to vocally respond (see response to primary concern above). This is now clearly stated in the method section (lines 505-509).

2. Normalized firing patterns shown for their neuronal population ranked by response onset show that about 2/3 of their neurons (interneurons as well as projection neurons) have short response latencies to both familiar and unfamiliar calls. The remaining third of the neurons have much longer onset response latencies for familiar calls but weak to no responses for unfamiliar calls. Is it possible that the classifier relies on the weight of this last set of neurons to discriminate between call types? Certainly, by eye, these long latency neurons show a striking difference in responsiveness to the different call classes.

We thank Reviewer #2 for this interesting observation. We appreciate the opportunity to test the hypothesis that our classifier relies solely on late-responding neurons. To address this, we conducted a supplementary analysis in which we split the neural activity into two distinct periods 1) during playback (0-100ms) and 2) after playback (100-400ms). Our analysis showed that using either of these timeframes individually resulted in worse classification accuracy compared to using the entire dataset (Fig 2e). Interestingly, when we used only the late responses, the classifier was unable to reliably predict which call was presented. Therefore, we conclude that the population activity across the entire timeframe originally analyzed (0-400ms), continuously integrates information about familiarity. We have included this supplementary analysis in the text (lines 245-250) and in a new Supplementary Figure S7 Fig.

3. Unless I missed it, the authors should provide data, maybe in the form of a UMAP plot, to show that familiar and unfamiliar calls have overlapping acoustic features and that birds (and HVC) are not simply discriminating between these two classes of calls by acoustics features but because of familiarity. This will be an important control to provide.

We thank Reviewer #2 for highlighting the need for additional consideration of acoustic features. Due to the limited data points available (one per playback variant), and that UMAP approaches are better suited for finding structure in large and high-dimensional datasets, we decided to use Principal Component Analysis instead. Specifically, we conducted PCA using the sound waveform time bins as features. This gave us 7 main PCs explaining 93.47% of the variance across playbacks. Projecting our playbacks into this PC space, we then determined the optimal number of clusters to understand whether these calls were categorized in the same cluster (indicating acoustic similarity) or in different clusters (indicating acoustic differences). We did this using the silhouette score, which suggested that two was the optimal number of clusters (n=2). We then used a k-means clustering algorithm to identify these two clusters. When we labeled the individual calls based on whether they were familiar, unfamiliar, or both, we found no systematic difference between the clusters (S3 Fig). This finding suggests that although the birds apparently perceive subtle differences between calls, they did not systematically discriminate between them based on categorical differences in their acoustic properties. This information has been added to the main text (lines 265-272) and methods (lines 513-521), and the PCA plot is now included in Supplementary Figure S3 Fig. For visualization purposes, we plotted playbacks using the first two principal components, which accounted for 51.03% of the total variance.

Reviewer #3: In this study, the authors investigate the neuronal correlates of call processing in a songbird species, the zebra finch. They first provide behavioural evidence that males zebra finches respond differently to calls emitted by familiar compared to unfamiliar conspecifics. Building on this, they then turn to large-scale electrophysiological recordings using Neuropixels probes. They focus their recordings on the HVC, a premotor area known for its critical role in the birdsong behaviour, but also involved in vocal turn-taking of zebra finches, as shown by a previous work from the same team. Here, electrophysiological recordings were made in awake head-fixed birds while they were exposed to a set of 5 distinct auditory stimuli: a call from a familiar female, a call from an unfamiliar male, a call from an unfamiliar female, a 20 kHz pure tone pulse and a silent playback. Based on the shapes of the isolated units, the authors were able to separate putative interneurons (INs) from putative projection neurons (PNs). Both INs and PNs had their spiking activity modulated by the familiarity of the calls. PNs exhibit lower level of responses compared to INs. This study thus shows that auditory stimuli conveying specific social information impact the neuronal processing within a premotor area.

Overall, I found the paper well-written and easy to follow. Results are clear and conclusions are in line with the data. Below are some comments/questions (not sorted by importance).

We thank Reviewer #3 for their positive feedback on our manuscript.

1. I wonder whether the authors could be able to provide some assessment of how familiar the familiar calls are, and how to ensure that the unfamiliar calls remain unfamiliar over the course of the study. Did you monitor the call interactions during the first five days of co-housing? If yes, on average, what is the number of call interaction per day or during the five days?

We thank Reviewer #3 for this interesting question. Based on previous literature [17, 18], pair bonding occurs relatively quickly within the first days of cohousing. Using this, we induced familiarity through a specific experimental setup (i.e. housing each male with a female for five a minimum of five days). We

did not, however, monitor the number of call interactions during this co-housing period. The unfamiliar calls were from individuals each tested bird had never encountered. While we cannot completely rule out that the birds became accustomed to the sound of these calls over the course of the experiment, it is unlikely they developed social familiarity with them, and at the very least they would always remain less familiar than the cage-mate call. The calls were presented to birds while tested in isolation, without a social partner with whom to directly associate the call. Nevertheless, our response to Reviewer #3's second comment (below), will also address the issue of repeated exposure to calls.

2. In fig 1d, I guess the results are a summary of the 4 test days. Did you notice a day effect in the behavioural responses? This might be critical for the unfamiliar calls since they may become familiar after many exposures.

To address the question of increasing call familiarity, we re-analyzed the behavioral responses for each of the four days of exposure. We observed that the overall vocal response probability decreased over time for both familiar and unfamiliar calls. However, the latency and variability of these responses remained stable. Crucially, despite these changes, the behavioral difference between responses to familiar and unfamiliar calls persisted throughout the experimental days. To examine whether the behavioral parameters (response probability, latency, latency variability) differed depending on familiarity and across days, we used a two-way repeated-measures ANOVA. We found a main effect of familiarity in all behavioral measures, and no interaction between familiarity and day, showing that the familiarity effect was not significantly influenced by increasing playback exposure. This indicates that the effect of familiarity was consistent across days (S1 Fig), and that even if the birds became more accustomed to the unfamiliar calls, their responses to the socially familiar calls were still distinct. We have now added this analysis to the Supplementary Figure S1 Fig and also included this finding in the main text (lines 107-109).

3. How distinct are the calls? It could be interesting to provide a measure of (dis)similarity between the different calls.

We have included an examination of the acoustic similarity of calls in response to Reviewer #2's question. Please refer to the updated information in the main text (lines 265-272 and 513-521) and in S3 Fig.

4. About the number of playbacks for each sound, it is written in the method section ("Behavioral experiment" section) that there are 20 blocks of 6 repetitions of each playback sound, during 4 days. So if I get it correctly, each sound has been played back 480 times (20x6x4). Why are there "only" 234 trials for Familiar calls, 232 for unfam call 1, 228 for unfam call 2 shown on fig 1c? Is it that only trials with a call response are shown?

We apologize for the confusion. As stated in Fig 1 caption (lines 132-134), the top panel in Fig 1c includes data from the first 2 experimental days to exemplify response tendencies (2 days=240 trials). This was done in this way to facilitate visualization, given the decreased response rates in later days, which are now explicitly analyzed (S1 Fig). On the other hand, all quantifications include all trials, for example Fig 1c bottom panel, d, e, f, and g. Still, the discrepancy in the number of trials in Fig 1c top panel (not 240) stems from the following exclusion criterion: we removed any trials where the birds responded with distance calls or songs. We have now added this exclusion criterion to the Methods section in lines 499-501.

5. In fig1d-f, the large error bars for behavioural responses to unfamiliar calls may suggest a difference between the response to the unfamiliar males vs unfamiliar females. It could be interesting to assess whether behavioural responses do differ between unfamiliar males/females. One suggestion is to show in a supplementary fig the same plot than in Fig1d-f separating unfamiliar females and males, and a statistical comparison between the two.

We appreciate this valuable suggestion from Reviewer #2 and agree that assessing behavioral differences in response to unfamiliar males vs. females would be interesting. We have separated our dataset accordingly and added a Supplementary Figure S2 Fig as suggested by the reviewer. We found that the birds responded differently when they interacted with familiar versus unfamiliar male calls. In case of responses to female calls only the response latency was modulated to a significantly different

extent for familiar versus unfamiliar female calls. This result is now added in the main text (lines 114-119).

6. Did you ever have responses with song production? If yes, was it sufficiently frequent to assess whether the amount of songs produced differ according to the call familiarity and/or sex of the caller?

We did observe instances of song production, but they were infrequent and primarily occurred with a particular bird (experiment id 9 below). Due to the very small sample size, we cannot reliably assess whether the number of songs produced differs based on the call's familiarity or the caller's sex. Nevertheless, we provide the different proportions of song responses that were given across the experiments (Reviewer Figure 2). In general, songs were produced in less than 2% of the trials for most birds, and the relative proportion of this response type doesn't show clear trends of variation depending on call playback id or sex.

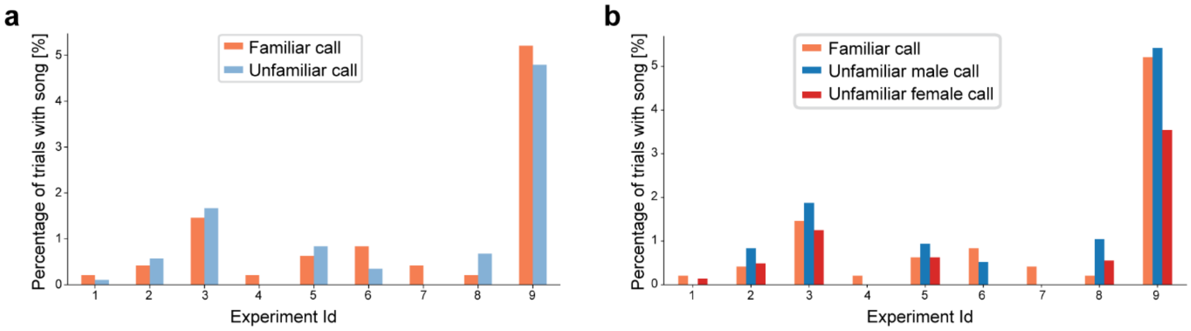

Reviewer Fig 2. Song responses to different call playbacks. (a) Percentage of trials with song responses for the different playback types. Percentages are normalized according to the total amount of trials per condition (n=9 experiments, birds=7, days=4). **(b)** Same arrangement as in a, but separating unfamiliar playbacks by sex.

7. Line 92-95: I do not understand why the accuracy level indicates that the inherent features of the acoustic signals are not so important in predicting the individual response. Could you please provide some more info?

We reason that the accuracy level does not rely on inherent acoustic features because the same acoustic signals were used for both familiar and unfamiliar conditions. For instance, a specific call might be familiar to Bird A, but unfamiliar to Bird B, and vice versa. This means that if the classifier's performance was solely dependent on responses to the acoustic properties of the sound, it should show accuracies closer to chance level, reflecting similar behavioral responses for each call playback regardless of the context. To add to this, we performed an additional assessment using Principal Component Analysis (PCA) and k-means clustering. Our goal was to determine if the calls were acoustically distinct from one another (S3 Fig). We found that when we plotted the calls based on their PC acoustic space, they did not sort into separate clusters (e.g., familiar vs. unfamiliar). Instead, the data showed no clear separation across contexts, indicating that although individual calls differ (which birds can evidently detect), acoustic features are unlikely to explain the behavioral differences we attribute to familiarity.

8. To provide support for the assumption made in the discussion on the modulation of PNs via a feed-forward inhibition from INs (lines 318-321), it would be interesting to take advantage of the simultaneous recordings of many units with the Neuropixels probes to compute some cross-correlation between PNs and INs, at different time lags regarding the call onset.

We appreciate this suggestion. We calculated cross correlation coefficients (per recording id) by computing each interneuron's average firing rate in a window around the presented playbacks (familiar and unfamiliar, time window=-100 to 400ms from playback onset). Each interneuron was compared to all corresponding (simultaneously recorded) projection neurons, and the time lag (in ms) with the highest correlation was taken. Then, we obtained the mean maximum correlation and corresponding time lag across all these interneuron-projection neuron pairs. This way, we could quantify the proportion of cases in which patterns of interneuron activity systematically precede projection neuron activity. We found

91/169 interneurons to be most correlated at a negative time lag, indicative of their activity preceding that of the projection neuron population. In addition, higher correlation values were obtained for negative time lags (Spearman correlation=-0.34, p= 7.2×10^{-6}), suggesting that interneurons exhibit tighter temporal coupling when their firing leads the projection neuron populations, and potentially actively shaping or setting up feed-forward inhibition. Results were added into Supplementary Figure S10 Fig, and into the manuscript in lines 272-279, 372-376 and in method lines 574-586.

9. It could be interesting to add a small paragraph in the discussion about whether the social modulation of neuronal activity elicited by the conspecific calls (familiar/unfamiliar) is an arising property of the HVC, or if it is inherited for upstream (auditory) areas. I'm thinking of NCM whose neurons do not discriminate familiar vs unfamiliar calls, except if there is an audience (Menardy et al, Eur. J Neurosci 2014).

We discuss our findings in light of this previous work in the discussion (lines 376-384).

10. Line 374-375: what are the 5 different playbacks/bird should be provided here (1 familiar female, 1 unfam male, 1 unfam fem, a 20 kHz pure tone pulse and a silent playback)

Each bird was presented in total with 5 different playbacks from a collection of 9 sounds that included a familiar (female) call, 2 unfamiliar male calls, 5 unfamiliar female calls, and a catch playback (silent period). From these, all female playbacks served as both familiar and unfamiliar for different birds. This information is added in lines 436-439.

11. Line 359: why vocalizations were overlaid with a 20 kHz pure tone pulse?

It was used to facilitate detection and separation of playbacks from vocal responses when analyzing recordings from soundboxes during the behavioral experiments. This is important as often a simple amplitude threshold does not provide reliable segmentation and playback onset detection. Then, using a stable 20kHz pure tone outside of zebra finches hearing range [54, 55] assures consistent playback onset identification and alignment of responses to stimulus. Furthermore, since we used this tone for behavioral experiments, we also added it to neural experiments, where it also facilitated playback onset detection.

12. Fig 2 & 3: it would be nice to see the waveforms of the exemplar units.

They are now added to the main Figures 2 and 3.

Minor comment :
13. Fig 1g : there are no “top right” and “bottom right” panels (but middle, and right).

This has been corrected in the manuscript.
