# Supplementary material for: Social familiarity strengthens neural and vocal responses to conspecific calls in zebra finches
Source: PLoS Comput Biol. 2026 Mar 11;22(3):e1014024. doi: 10.1371/journal.pcbi.1014024 (PMC12978450; doi:10.1371/journal.pcbi.1014024)
Supplement: Attachment — Submitted filename: Rebuttal_Gomez_Revision2_final.pdf [file pcbi.1014024.s015.pdf]

Reviewer #1: The authors have done a wonderful job in addressing my concerns. I support the publication of the manuscript in PLOS Computational Biology.

We would like to thank Reviewer #1 for their support and constructive feedback.

Reviewer #2: I thank the authors for their careful reworking of the text to address the reviewers's comments. I also appreciated extra analyses they provided to investigate potential confounds.

I am happy with the way they addressed my comments and am now happy with this manuscript.

We appreciate the positive assessment of our work by Reviewer #2.

My two very minor comments are:

(1) It might be worth providing a bit more explanation in the legend of Supplemental Figure 3b as it took me a while noticing there we two clusters, because the dot in the bottom right is really easy to miss.

We now specifically point out the single data point in our cluster in the Figure legend accompanying Supplemental Figure 3b.

(2) I would have like to see a bit more hand holding for supplemental Figure 7. The point, for me at least, is not entirely obvious that 0-100 sec is significant whereas analysis of 100-400 sec is not. This could be made more explicit in the figure perhaps as well as in the description of the figure legend.

We agree with Reviewer #2 and have now added a more detailed description of this analysis in the Figure Legend of Supplemental Figure 7. Additionally, we provide an interpretation of this result in the main text (lines 251-253).

Reviewer #3: I would like to thank the authors for their revised version of the MS. They have properly addressed all my comments.

We thank Reviewer #3 for their support.

I have a few minor comments:

- on fig 2f, since the correlation between unfam/fam max FR is not significant ($p=0.051$), there should be no red and black lines.

We eliminated red and black lines for the mentioned plot.

- please add the unit of the firing rate in the legend of figs 2 & 3 (lines 188-189 & 216-218)

Added.
